# Early virtual reality adopters in Spain: sociodemographic profile and interest in the use of virtual reality as a learning tool

**DOI:** 10.1016/j.heliyon.2019.e01338

**Published:** 2019-03-14

**Authors:** Roberto Sánchez-Cabrero, Óscar Costa-Román, Francisco Javier Pericacho-Gómez, Miguel Ángel Novillo-López, Amaya Arigita-García, Amelia Barrientos-Fernández

**Affiliations:** a Department of Social Sciences and Applied Languages, Alfonso X el Sabio University, Madrid, Spain; b Department of Languages and Education, Nebrija University, Madrid, Spain; c Department of Geography and History, National Distance Education University (UNED), Madrid, Spain

**Keywords:** Education, Computer science, Sociology

## Abstract

This study describes the social and demographic profile of the first generation of users of marketed virtual reality (VR) viewers in Spain and, subsequently, it assesses the interest in its use as a learning tool. For that purpose, an online questionnaire created *ad hoc* was administered to a sample of 117 participants. The relationship between twelve variables was analysed comparing means through the *Snedecor's F* distribution and the contingency tables through the *Chi-squared* test and *Somers' D*. Among other issues, it was concluded that the virtual reality user profile at present corresponds to a person older than 36, mainly men, with higher education and having acquired their viewer no longer than one year ago. Concerning the interests of virtual reality users as a learning tool, only a few of them currently use virtual reality for this aim, but they mainly show an interest in using the virtual reality as a learning method and they feel optimism regarding the future use of this technology as a learning tool. However, this is not the case among users of video game consoles (*PSVR*), who are mainly men not interested in their use as a learning tool at present. Finally, it can be stated that current use as a learning tool among teachers and students is occasional and preferably via smartphones.

## 1 Introduction

Even though at present there is a close relationship between computer science and virtual reality (and therefore it would seem impossible to understand and achieve the latter without the former), as a theoretical conceptualization, it is possible to establish an early origin of virtual reality rather much before the beginning of computer science. This fact shows how ancient the desire of the human being to create and experience realities alternative to the physical reality is (Artaud, 1938–1958; Heilig, 1962; Kavanagh et al., 2017).

This brief curiosity shows that virtual reality is not another technological advance that someday will fall into disuse, but rather it certainly is the greater technological revolution of our generation. It actually responds to a human desire that goes beyond computer environments and artificially created graphics (Hockley, 1997; Dombrowski and Dombrowski, 2017). It implies the fact of living an experience beyond the physical reality itself and being capable of making everything that can be imagined to come true: historical events, places of fantasy, imagined events, etc (Costa-Román, 2016; Sánchez-Cabrero et al., 2018a,b). Therefore, human beings expand the boundaries of their creations, experiences and learnings to everything that can be represented into a virtual environment. This, in fact, has many fewer restrictions and is closer to the real experience than any other device has designed until now (Steuer, 1992; Cohen-Hatton and Honey, 2015; Gadelha, 2018).

The scientific field cannot stand aside of this technological revolution. It must lead the advances and show the possibilities of this technology in the different fields of interaction between technology and human beings (Vivanco and Gorostiaga, 2017). This is the context that frames this research. It is based on a first approach by the authors to the practical use of virtual reality in primary classrooms in Spain (Costa-Roman, 2016) and the exploration of the existing possibilities in terms of applications and software for the different current commercial viewers (Sanchez-Cabrero et al., 2018a,b). A great interest of learners towards the use of this technology was clearly observed, presenting a great intrinsic motivation in its use. It was verified that, not only the virtual reality offers great possibilities for teaching at many levels, but it also is a relatively unexplored area in its beginnings that urgently needs to deepen its application in the classroom. There is a whole new generation of students and learners who will naturally learn and interact within the digital environment. Furthermore, their communicative style needs tools and applications adapted to their learning style (Wu and Kuo, 2017). For this reason, it is especially important to lay the foundations of both (1) the state of play and (2) the interest that this technology arouses in its own users as a learning tool (Pan and Hamilton, 2018).

Taking as a reference the definition of virtual reality found at the *Encyclopaedia Britannica*, created by Lowood (2015), virtual reality is understood as the immersion of an individual in an artificial environment, generally created using computer means, which simulates a complete reality for the users and enables them to interact with this environment to a certain degree. The *‘being there’* feeling is a *sine qua non* condition to achieve a virtual reality experience. This definition sets the limit of what virtual reality is; *vis-a-vis* what it intends to be but without becoming it. Virtual reality requires that the users feel the immersion in a reality, other than the physical one, where they may have a certain degree of interaction. When the feeling of ‘journey’ towards another reality is not experienced, there is no virtual reality at all; thus, a 3D video experience is not virtual reality because the user does not feel that needed dissociation between mind and body. The use of video games is not considered virtual reality either, even though several studies from the 90s referred to it with experiences that did not fulfil minimum requirements (Rizzo and Koenig, 2017).

From the beginning of the computer science maturity, the possibility of simulating artificial reality environments created with computer means started to be envisaged. However, none of them could properly reflect an actual artificial reality due to lack of technological means in each period (Brooks, 1999; Zyda, 2005). In order to make an individual feel that he/she is inside another reality, virtual reality requires the use of perceptive devices similar to the human senses. Currently, these human senses are still more complex than the technology designed until now (Sanchez-Cabrero et al., 2018a,b).

It is in this decade when the minimum requirements to make it possible have started to be glimpsed (Menzies et al., 2016). Until then, every experience had simulated three-dimensional environments rather complex, but they had not achieved a minimal immersion of the user in an artificial reality (Wu et al., 2015). Due to the limitations of technology itself, the participant did not perceive dissociation between mind and body in the artificially designed experiences. This dissociation is necessary to get the sensation of ‘*being there*’ proper to immersion in an artificial reality alternative to the physical one, and therefore, the desired virtual reality was not achieved.

Oculus' project (Oculus VR, 2012) could be considered as the pioneer in the development of virtual reality experiences with minimum requirements. It started with a *Kickstarter* project in 2012 and then in 2013 marketed its first development kit, establishing the first patent in the United States in 2014 (Luckey et al., 2014). From then, virtual reality has been seen as something possible but required some highly demanding specifications for current technology (Menzies et al., 2016; Domingo and Gates Bradley, 2017).

Current innovations in technology, computer science and optics have achieved that this idea, which other generations could only dream about, becomes now a reality, although within the limitations that exploring a completely new and until now undiscovered field means (Wu et al., 2015; Kim et al., 2017).

Before publishing its first commercial version in 2016, the VR Oculus project presented two virtual reality development kits in 2012 and 2014 that were among the first viewers with a real capacity to present an artificial reality that guaranteed minimal immersion. Other important projects were added later to that commercial version the same year, such as the HTC Vive or the PlayStation VR by Sony. The following year, other commercial viewers started to be marketed, such as those sponsored by Microsoft under the acronym WMR (Windows Mixed Reality), thus increasing the virtual reality commercial offer that was available for the average user. Therefore, the period that has elapsed between the first virtual reality viewers that guaranteed an actual virtual reality and the current period has been rather brief. Within this brief period of less than two years, users of virtual reality have been able to try this new technology and have acquired knowledge regarding its real application, which is essential in order to determine the practical and daily use of this technology.

Due to the fact that commercial virtual reality viewers have been on the market for less than two years, this technology is still far from settling into the general population. Therefore, it is difficult to know what society will ask of this technology when it becomes mainstream, as observed by Mütterlein and Hess (2017). The interests shown by early adopters offer us the keys to glimpse that moment and estimate the extent of their demands. We will be able to advance the development of the necessary applications or to take the necessary measures to meet them, in the same way that the so-called 'beta testers' do with computer software and videogames (Stavova et al., 2018).

The results offered by this study would therefore be a first step towards a better understanding of the relationship between human beings and virtual reality. As Pan and Hamilton (2018) state, VR currently still raises many questions, especially from the point of view of a learning tool. Subsequent scientific research may focus on adapting its interventions and experience design to the observations of this study, optimizing the effectiveness and appropriateness of its intervention.

There are previous studies that support research in relation to the acceptance and attitudes generated by virtual reality in society today. For example, Disztinger et al. (2017) and Tussyadiah et al. (2018) explored the acceptance of virtual reality for tourist use. They conclude that knowing and promoting different tourist sites was seen in an optimistic and highly valued way. In a completely different field, Dockx et al. (2017) showed that even elder population could develop positive attitudes and high satisfaction with the practical use of virtual reality.

One of the most important potential use of VR, among others, is found in the educational field. The potential of a learning tool foreseen by a student when he/she is exposed to a factual experience of an almost real learning content, might suppose a much more solid, fast and effective learning than other more traditional learning situation. For instance, Fernández-Robles (2016) shows the way current primary students have a high motivation to learn using alternative reality elements, such as augmented reality, which reflects a very positive attitude from part of students to the inclusion of the VR in classrooms. Greenwald et al. (2017), on the other hand, lists the possibilities of VR for the development of collaborative learning. Bacos and Carroll (2018) show how the sense of presence influences the development of learning through virtual reality. However, there are also current studies against the fact of virtual reality's representing a significant improvement over poorer means of presentation. Leder et al. (2019) showed similar results and significantly lower costs using slide presentations, so it would be necessary to determine in further research the circumstances in which virtual reality can truly mark an improvement in learning.

Many educational situations could be enriched, accelerated or deepened through virtual reality, for instance, understanding a historical event by recreating its entire world from within, making the learner a part of it; recreating extinct species or environments and perceiving their real dimensions in contrast to today's world; travelling through unreachable places such as the universe or the deep sea; or even putting the learner in someone else's place to know how they feel. All these situations can contribute to the development of empathy and education in values, among others. These facts suggest that pedagogy should not be excluded from this technological revolution (Fowler, 2015; Kavanagh et al., 2017; Gadelha, 2018; Parong and Mayer, 2018).

Early adopters of the first commercial viewers of virtual reality could perform a first approach to the potential use as a learning tool, because they offer real information about their use, their interests and their possibilities (Buń et al., 2017; Moro et al., 2017; Nissim and Weissblueth, 2017; Lin et al., 2017; Yildirim, 2017; Yildirim et al., 2018). Usually, early adopters are a proven source of information for the settling of new technologies. However, in virtual reality, their participation is essential, since the possibilities for teaching and learning this technology cannot be observed from the outside and only those who cross the threshold of virtual reality are able to perceive all its possibilities. Moreover, virtual reality viewers are in their early stages in the commercialization in the market. This fact gives a great scientific and practical value to this study, as the data obtained have not been previously consulted and could easily serve to glimpse the future of the relationship between virtual reality and the educational field.

The present study aims to collect this information through the administration of a questionnaire designed to assess and value the experiences lived by the early adopters of virtual reality in Spain with viewers that guarantee a sufficient immersion, such as those described before. It is intended to assess the interest of use of this technology as a learning tool in a near future, reflecting the early adopters' interests and privileged points of view.

With the purpose of finding answers and confirmations to the mentioned questions, this research sets out two objectives: (1) To describe the social and demographic profile of early adopters of virtual reality viewers in Spain and (2) to assess the interest that these users may have in its use as a learning tool in order to be able to evaluate its possible future inclusion within the formal educational setting.

## 2 Material and methods

### 2.1 Participants

Considering that the first virtual reality viewers have been on the market for less than two years and the settling of technology in society is still limited (do not confuse the settling of technology with its popularity: virtual reality is very popular, but a large part of society has never tried it), the population from which to extract a scientifically acceptable sample is very small.

It is very difficult to know the exact number of early adopters of virtual reality in Spain, given that most manufacturers do not make their sales public so as not to discourage future investors and users. According to indirect sources (Statista, 2018), it is estimated that by 2018, less than 4 million viewers were sold on the world market. This represents a percentage of technological applications, software and video games users significantly lower than 1% of the total population (Newzoo, 2018), which approximately is 42% of the total population of society (Enterteinment Software Association, 2016). Therefore, it can be considered that currently, less than 5 per thousand of the population are of early adopters.

Another problem addressed in the study is how to locate virtual reality users, since it is a heterogeneous group with different interests and socio-demographic profiles. They are distributed according to different viewers (PSVR, Oculus Rift, HTC Vive, WMR, etc.) and platforms (PC, PS4, smartphones, etc.), so it will be necessary to access those global online portals they usually visit searching for information and help to properly use their viewers and be informed.

The *elotrolado.net* is a forum portal for new technologies, digital leisure and video games with more than 460,000 users and 10 million visits per month (Similarweb, 2018). It is the only Spanish-speaking portal that has a specific virtual reality forum with more than 400 threads and 76,000 early VR adopters' messages/contributions from all viewers and platforms on the market (elotrolado.net, 2018). Therefore, it is the ideal place to obtain a representative sample of early VR adopters.

On February 15, 2018, a new thread was created in the virtual reality forum explaining the study to be carried out and hosting a hyperlink to the questionnaire designed and maintained through the private server encuestafacil.com. This thread received 1,000 visits from users of elotrolado.net.

The sample of this study consists of 117 virtual reality users (21 women and 96 men) owing any of the viewers marketed in Spain, with an average age of 36.91 years old (36.19 for women and 37.07 for men) and a standard deviation of 6.39 (7.50 for women and 6.15 for men). The sample was obtained after filtering 578 open questionnaires and eliminating the 'undelivered' cases and 36 incomplete questionnaires.

### 2.2 Instruments for obtaining data

An *ad hoc* online questionnaire was designed and hosted at the *Encuestafacil.com* private server, so that participants could remotely have access from any kind of electronic device with Internet. The questionnaire was assessed by the *Scientific and Ethical Committee of the Nebrija University* and overcame a severe validation process led by external experts. Moreover, the designed questionnaire had a high reliability and internal consistency, measured through the Alpha's Cronbach (0.826).

This questionnaire consisted of a first page where the participants expressed their written and informed consent, three more pages with 25 quantitative questions and a fifth page with open questions for future qualitative analysis.

Concerning this study, only quantitative results obtained in questions related to demographical aspects and possibilities of use as a learning tool of the technology have been considered in the analysis.

### 2.3 Assessed variables

The following variables were taken into account for the purposes of this study:

1. Gender: As a dichotomous variable (man or woman). Although gender differences in the use of video games and new technologies are narrowing, current studies such as those by Rutherford (2018) or Dindar (2018) show that this is still an attributive variable to be considered in this type of studies.
2. Age: As a discrete quantitative variable.
3. Educational level: As an ordinal categorical variable with four options (primary, secondary, university and postgraduate).
4. Current direct relationship with formal education: As a dichotomous variable (YES, if the participant is a teacher or a student, and NO, if he/she is none of them). This variable is important, as its daily involvement in formal education can influence decisively the interest of the virtual reality user in its use as a learning tool.
5. Previous experiences with advanced virtual reality viewers: As a dichotomous variable (YES, if the participant has previously used a viewer special for video game consoles or personal computers, and NO if he/she has only used mobile phones in order to experience virtual reality). This variable is important due to the limitations of the possibilities of mobile experiences, and users who have not tried the advanced VR might not see its full potential.
6. Level of the private viewer: As an ordinal categorical variable with three options (mobile phone, video game console and personal computer). This variable reflects different uses and qualities according to the type of user. The PC user has more possibilities, the mobile user wants an easier access and the video console user is mainly interested in leisure.
7. Number of years using virtual reality: As an ordinal categorical variable with four options (Less than one year, Between one and two years, Between two and three years and More than three years). This variable is important, as users using development kits for more than two years started with much more expensive kits, less advanced and much more exclusive, demonstrating a true passion for VR. Additionally, a descending curve of interest based on the antique technology could reflect that virtual reality can generate weariness, tiredness or frustration in the long term.
8. Frequency of use: As an ordinal categorical variable with four options (Occasionally, Once a week, Several times a week and One or more hours each day).
9. Current use of virtual reality as a learning tool: As a dichotomous variable (YES, if the participant uses virtual reality in order to learn and obtain knowledge, and NO, otherwise).
10. Interest in the use of virtual reality as a learning tool: As a dichotomous variable (YES, if the participant shows an interest in its educational use, and NO, otherwise).
11. Interest in the future use of virtual reality in formal education: As a dichotomous variable (YES, if the participant would like to learn through the use of this tool, and NO, otherwise).
12. Optimism regarding the future pedagogical possibilities of virtual reality: As a dichotomous variable (YES, if the participant considers that the educational field will have a proper development within the virtual reality sphere in the future, and NO, otherwise).

### 2.4 Design and procedures

A cross-sectional descriptive study of virtual reality users in Spain and the interest in the use of this technology as a learning tool assessment of the technology was carried out, evaluating the influence and interaction of several nominal, ordinal and quantitative variables.

Once the data of the participants was anonymously collected, the statistical analyses were performed using SPSS statistical software.

Arithmetic mean and standard deviation were used as descriptive statistics for the quantitative variable ‘Age’, and frequency distribution was used for the rest of variables. Concerning inferential statistics, the significance level of the discrete quantitative variable ‘Age’ has been analysed by comparing means through *Snedecor's F* distribution non-considering equality of variances. For the rest of the nominal and ordinal variables, as purely quantitative analyses could not be carried out, *Chi-squared* test was conducted on contingency tables to test whether or not a relationship exists between variables, and *Somers'D* to reflect strength and direction of the association between variables.

Finally, decisions regarding the significance were made with confidence levels of 99% (α: 0.01) and 95% (α: 0.05) in the collected results.

## 3 Results

Table 1 presents the frequency distribution obtained through the studied sample in the nominal/dichotomous variables and in the ordinal variables assessed during the study.

**Table 1:** Frequencies distribution according to the variables considered in the study.

**Gender**	**Frequency**	**Percentage**
Man	96	82.1
Woman	21	17.9
**Educational level**	**Frequency**	**Percentage**
Primary	3	2.6
Secondary	39	33.3
University	49	41.9
Postgraduate	26	22.2
**Current direct relationship with formal education**	**Frequency**	**Percentage**
None	90	76.9
Teacher or student	27	23.1
**Previous experiences with advanced virtual reality viewers**	**Frequency**	**Percentage**
No	21	17.9
Yes	96	82.1
**Level of the private viewer**	**Frequency**	**Percentage**
Mobile phone	26	22.2
Video game console	54	46.2
Computer	37	31.6
**Number of years using virtual reality**	**Frequency**	**Percentage**
Less than one year	72	61.5
Between one and two years	35	29.9
Between two and three years	4	3.4
More than three years	6	5.1
**Frequency of use**	**Frequency**	**Percentage**
Occasionally	43	36.8
Once a week	25	21.4
Several times a week	40	34.2
One or more hours each day	9	7.7
**Current use of virtual reality as a learning tool**	**Frequency**	**Percentage**
No	101	86.3
Yes	16	13.7
**Interest in the use of virtual reality as a learning tool**	**Frequency**	**Percentage**
No	84	71.8
Yes	33	28.2
**Interest in the use of virtual reality in formal education in the future**	**Frequency**	**Percentage**
No	57	48.7
Yes	60	51.3
**Optimism regarding the future pedagogical possibilities of virtual reality**	**Frequency**	**Percentage**
No	62	53.0
Yes	55	47.0
**Total**	**117**	**100.0**

In a first simple analysis of the results, Table 1 shows that the majority options are the following: to be a man (82.1%); to have at least university studies (university students plus postgraduates amounts to 64.1%); not to have a direct relationship with education (76.9%); to have previously tried advanced virtual reality viewers (82.1%); to be users of video game consoles viewers (46.2%); to have acquired a viewer during the last year (61.5%); to use virtual reality at least once a week (63.2%); not to use virtual reality as a learning tool (86.3%); not to have interest in its use as a learning tool (71.8%); to have interest in learning through virtual reality in the future (51.3%); and not to have optimism regarding its future pedagogical possibilities (47%).

**Fig. 1:**
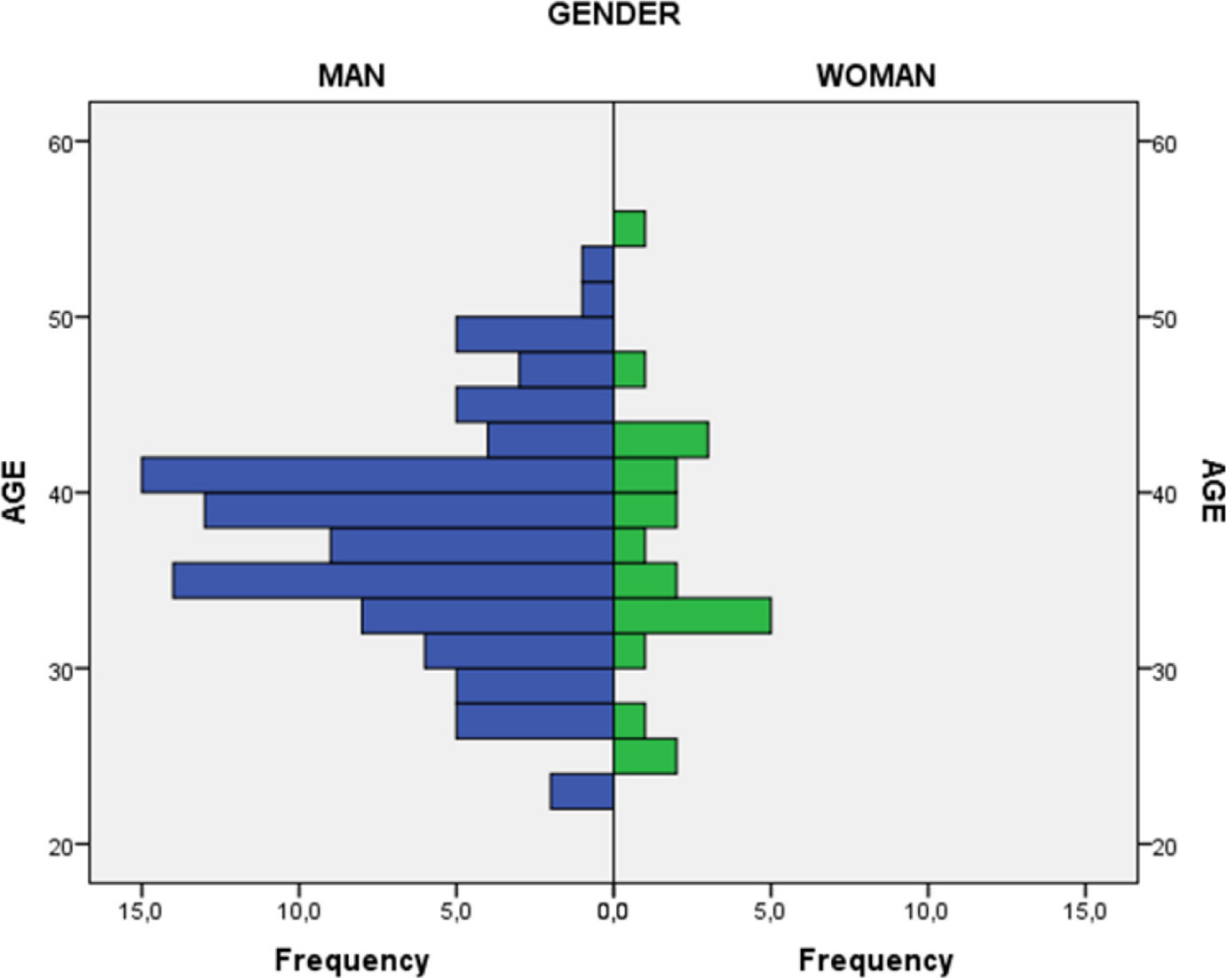
Age and gender pyramid.

Source: Self-elaboration

Concerning the discrete quantitative variable ‘age’, the global mean obtained was M: 36.91 with a standard deviation of SD: 6.39. In relation to its combination with the “gender” variable, it can be observed in Fig. 1 below that presence of men (82.1%) is significantly higher than presence of women (17.1%). However, age difference does not imply statistical significance according to gender, as it can be seen in Table 2. Only the ‘Optimism regarding the future pedagogical possibilities of virtual reality's variable presents a different performance in combination with the ‘age’ variable. Those who feel more optimistic are significantly younger (*M: 35.56; SD: 5.74*) than those who do not feel that way (*M: 38.11; SD: 6.74*).

**Table 2:** Comparison of means by age over the rest of the variables through ANOVA test.

Variables	Sum of squares	df	Root mean square	F	Sig.
Gender	13.418	1	13.418	.327	.569
Educational level	165.879	3	55.293	1.367	.256
Current direct relationship with formal education	20.616	1	20.616	.503	.480
Previous experiences with advanced virtual reality viewers	27.568	1	27.568	.673	.414
Level of the private viewer	161.535	2	80.768	2.013	.138
Number of years using virtual reality	169.738	3	56.579	1.400	.246
Frequency of use	57.568	3	19.189	.464	.708
Current use of virtual reality as a learning tool	51.353	1	51.353	1.261	.264
Interest in the use of virtual reality as a learning tool	33.517	1	33.517	.820	.367
Interest in the use of virtual reality in formal education in the future	4.044	1	4.044	.098	.754
Optimism regarding the future pedagogical possibilities of virtual reality	189.408	1	189.408	4.792	.031^a^

a Comparison of means is significant at the level of 0.05.

The significance of the observed differences between the various nominal and ordinal variables of this study may be analysed from Table 3. This table shows the values obtained from the contingency tables using the statistical *Chi-squared test*, which shows the significance of the correlations between two variables, and the *Somers' D*, which shows the significance and direction of the correlations observed.

**Table 3:** Contingency table using the chi-squared test (first value in each cell) and Somers' D (second value in each cell).

	Gender	EL	DRFE	PEV	LPV	YUV	FU	CUL	ILT	ILE	OFP
Gender	-	14.55** .3**	12.38** .32**	20.6** −.42**	30.29** −.17	10.06* −.083	27.1** −.35**	18.463** .395**	1.24 .1	.352 .053	.177 −.038
EL	14.55** .3**	-	15.32** .3**	6.70 −.17*	13.63* −.17	15.37 −.02	17.45* −.26**	3.62 .14	.25 .03	3.99 .1	3.2 .11
DRFE	12.38** .32**	15.32** .3**	-	12.38** −.32**	22.57** −.31**	5.11 −.06	8.04* −.18*	4.46* .19	1.35 .11	.138 −.03	.018 .0.12
PEV	20.60** −.42**	6.7 −.17*	12.38** −.32**	-	59.88** .47**	1.56 .0.8	17.82** .28**	4.81* −.2	.33 −.05	.012 −.01	.82 .08
LPV	30.29** −.17	13.62* −.17	22.57** −.31**	59.88** .47**	-	12.02 .05	31.92** .3**	19.07** −.09	2.35 −.05	.64 −.03	2.06 .11
YUV	10.06* −.08	15.37 −.02	5.11 −.06	1.56 .0.76	12.02 .05	-	23.39** .16	18.18** .05	6.35 .09	2.88 −.081	5.25 .179*
FU	27.1** −.35**	17.45* −.26**	8.04* −.18*	17.82** .28**	31.92** .3**	23.39** .16	-	2.98 −.04	3.44 .13	7.296 −.044	2.957 .142
CUL	18.46** .39**	3.62 .14	4.46* .19	4.81* −.20	19.07** −.09	18.18** .05	2.98 −.043	-	32.18** .51**	4.17* .18*	3.52 .16
ILT	1.24 .1	.25 .0.3	1.35 .11	.33 −.05	2.35 −.05	6.3 .09	3.43 .13	32.18** .51**	-	11.02** .31**	5.1* .21*
IUE	.35 .05	3.99 .1	.14 −.03	.01 −.01	.64 −.03	2.88 −.08	7.3 −.04	4.17* .18*	11.02** .31**	-	10.62** .3**
OFP	.18 −.04	3.2 .11	.02 .0.12	.82 .08	2.06 .11	5.25 .18*	2.96 .14	3.52 .16	5.1* .29*	10.62** .3**	-

EL: Educational level; DRFE: Current direct relationship with formal education; PEV: Previous experiences with advanced virtual reality viewers; LPV: Level of the private viewer; YUV: Number of years using virtual reality; FU: Frequency of use; CUL: Current use of virtual reality as a learning tool; ILT: Interest in the use of virtual reality as a learning tool; IUE: Interest in the use of virtual reality in formal education in the future; OFP: Optimism regarding the future pedagogical possibilities of virtual reality.

*Correlation is significant at the level of 0.05/** Correlation is significant at the level of 0.01.

It should be taken into account that in order to establish a direction in significance, some nominal variables were given different scores, thus becoming ordinal variables. Therefore, the relationship between the man/woman category and the rest of the assessed variables can be easily observed. Otherwise it would not be possible to establish which gender has a direct or inverse association with the rest of the measured variables. The same conversion was carried out in every dichotomous variable, giving the higher score to the category associated with the answer “YES” in each one of those variables.

From the previous *Chi-squared test* and *Somers´D* conducted on the contingency table, it can be outlined that some combination of variables shows a statistically significant relationship. For instance, the ‘gender’ variable shows that women have a significantly higher educational level, higher number of women are related to formal education and higher number of women use virtual reality as a learning tool. On the other hand, men have more frequently tried the advanced viewers and used virtual reality viewers.

The current direct relationship with formal education is also significantly and directly associated to the educational level and significantly and inversely associated to the fact of having tried an advanced virtual reality viewer, to the level of the private virtual reality viewer and to the frequency of use of virtual reality.

In addition to the before mentioned significant relationships, the frequency of use is significantly and directly associated to the fact of having tried an advanced virtual reality viewer and to the level of the private virtual reality viewer. The same variable is significantly and inversely associated to the educational level.

Another strong and direct significant relationship is found between the fact of having tried an advanced virtual reality viewer and the level of the private viewer.

Regarding the variables directly related to the use and interest of virtual reality as a learning tool, it can be generally observed that strong and positive significant correlations exist. Both affirmative answers of having interest in the use of virtual reality as a learning tool and in learning through virtual reality in formal education in the future are significantly and directly associated between them. They are as well associated with the two other variables considered (the current use of virtual reality as a learning tool and the optimism regarding the future pedagogical possibilities of virtual reality).

**Fig. 2:**
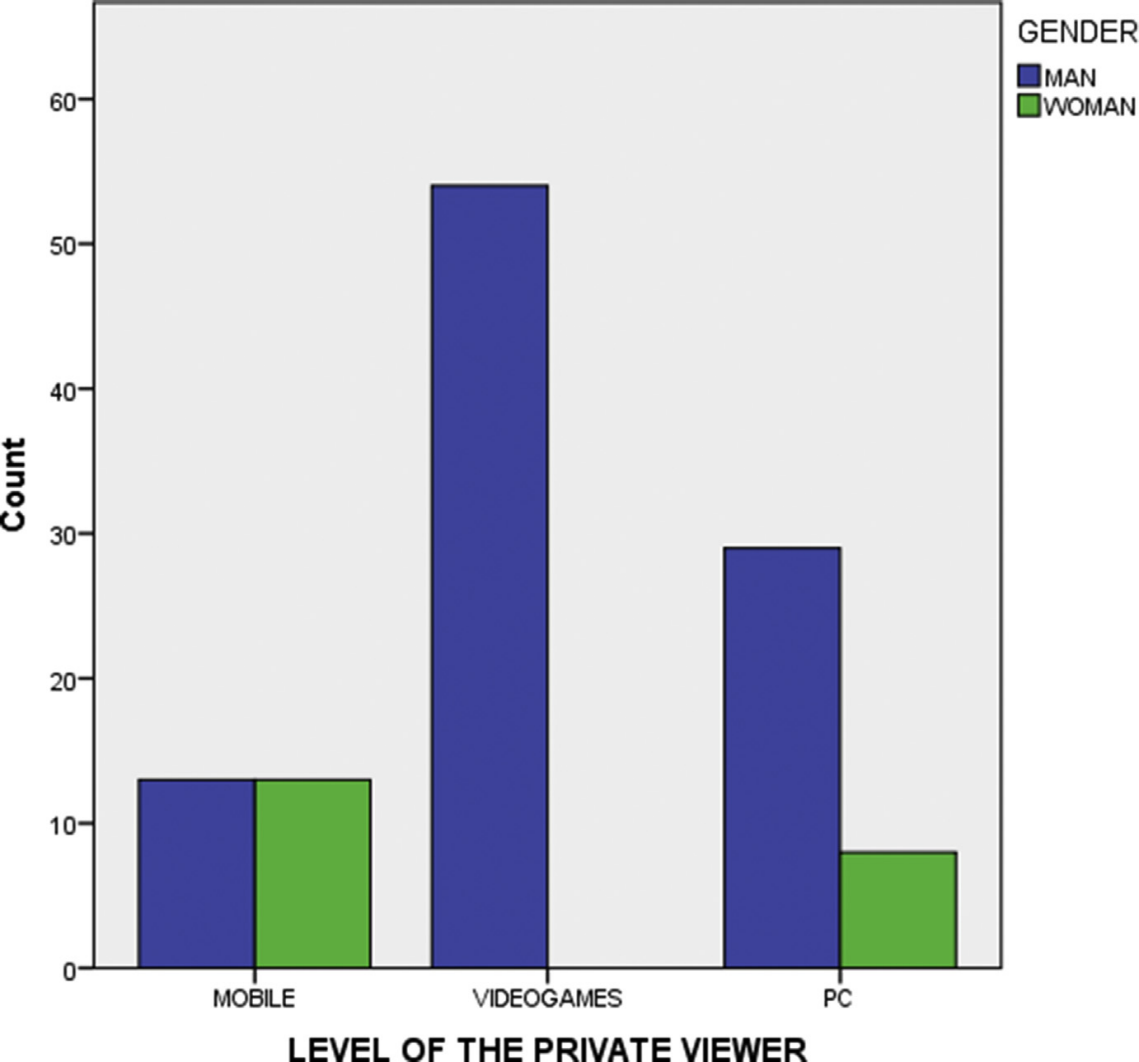
Level of the private viewer and gender.

Source: Self-elaboration

**Fig. 3:**
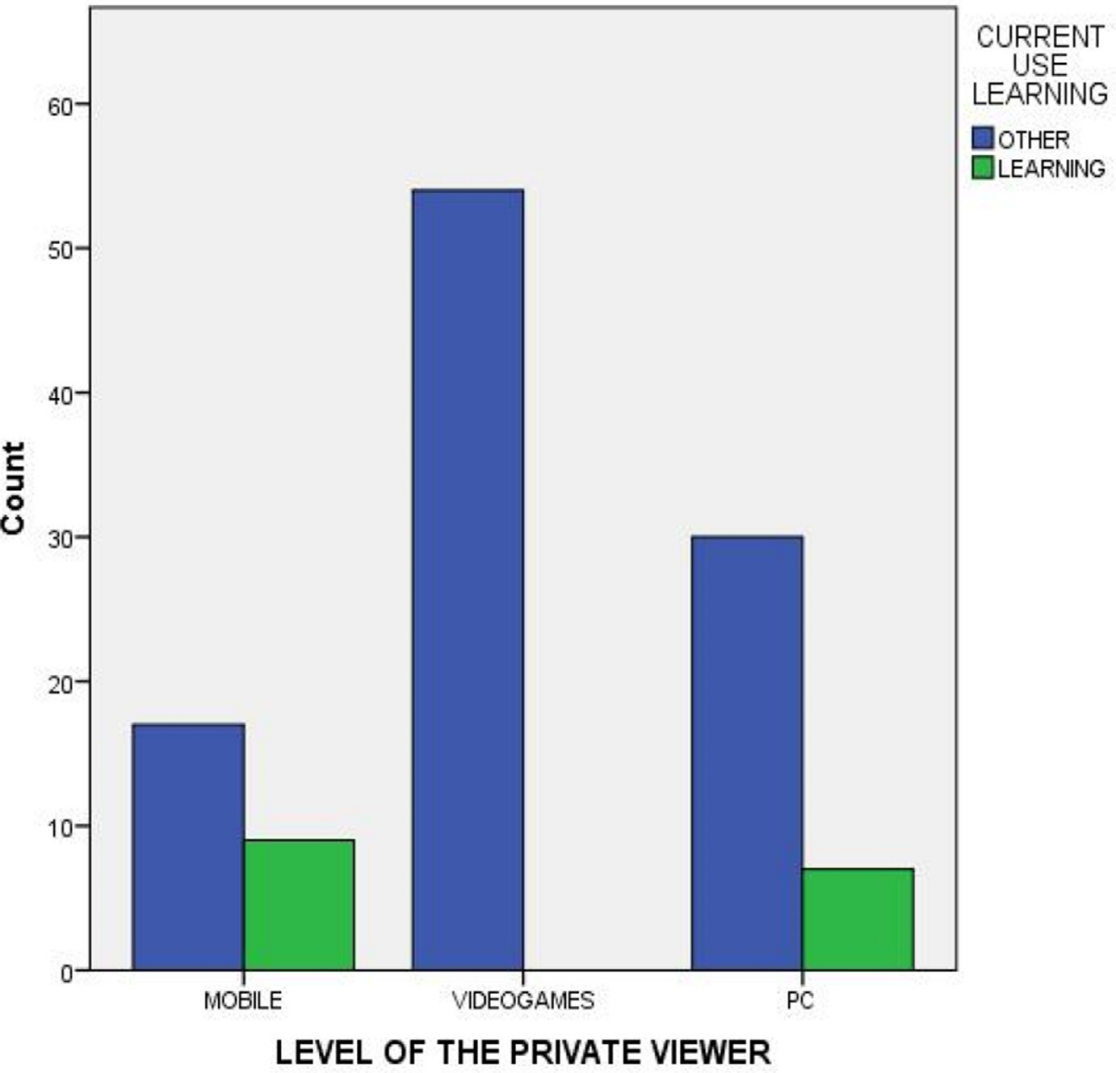
Level of the private viewer and current use.

Source: Self-elaboration

Results in the previous contingency table also show a statistically significant and nonlinear or second-degree association, which implies combinations of variables with significant values through the *Chi-squared test* but not through *Somers' D*. This situation exists when some of the categories for a variable produce a partial influence over other variable, such as ‘Number of years using virtual reality’ variable; its performance is different depending on the associated variable because in the case of categories in which users have been using virtual reality since recently, the interest of the user in this technology is still in a stage of discovering of its possibilities, so their frequency of use is unusually high and their interests very varied when wanting to try all the possibilities of the new technology. Regarding the ‘Level of the private viewer’, the category ‘Video game console’ has a different performance in relation to the ‘gender’ variable (see Fig. 2), and in ‘Current use of virtual reality as a learning tool’ (see Fig. 3). The fact that among the users of game consoles viewers (Sony PSVR) there are no women, nor interest in the use of the VR as a learning tool, indicates a very specific user profile with an interest directly associated with leisure.

## 4 Discussion

The description of the social and demographic profile of the first generation of virtual reality viewers users in Spain is the primary objective of the presented work. In a first analysis, this profile has been asserted, according to the percentages produced by this study, It would mainly correspond to a man (81.2%), with an age of 37, with university studies (62.1%), without a current direct relationship with formal education (23.1%), who had previous experiences with advanced virtual reality viewers (82.1%), mainly owner of a video game console (46.2%), who acquired a viewer during the last year (61.5%) and uses its own viewer at least once a week (63.2%).

Once carried out an in-depth analysis, the study of the contingencies between the evaluated variables led to the following conclusions:

• First of all, concerning the age, it should be remarked that the early adopters of virtual reality in Spain does not meet the standard profile of the first Information and Communication Technology (ICT) users (Yildirim, 2017), as the average age (M: 36.91) shows that those who are initially more interested in this technology are individuals closer to maturity than to adolescence. This could be certainly explained because this technology is currently expensive and shows little appreciation among the parents of teenagers. Therefore, an economic independence would be necessary and, as a consequence, many adolescents and young possible users cannot afford to access it in many cases. This fact contrasts with the data collected under the last survey about equipment and use of ICT at Spanish households in 2017 (INE, 2017). The main conclusion of this study was that individuals who used the mobile phone during the last three months (99.0%), who have ever used the computer (97.5%) and who have ever used the Internet (98%) correspond to the age group including ages between 16 and 24, closely followed by the age group between 25 and 34 years old and, in the third place, the age group of individuals between 35 and 44 years old (who in turn are those who stand out in this study concerning the use of virtual reality viewers (Table 4).

**Table 4:** Use of ICT devices according to demographic features and type of device.

	Individuals who used the mobile phone during the last 3 months	Individuals who have ever used the computer	Individuals who have ever used the Internet
Age: Between 16 and 24	99,0%	97,5%	98,0%
Age: Between 25 and 34	98,8%	93,9%	97,4%
Age: Between 35 and 44	99,2%	92,3%	96,7%
Age: Between 45 and 54	97,5%	85,3%	91,5%
Age: Between 55 and 64	94,4%	69,4%	76,1%
Age: Between 65 and 74	85,3%	45,7%	46,5%
• Regarding the ‘gender’ variable, both genders have a different profile and show distinct approaches towards virtual reality. Women who use virtual reality have a higher educational level, are often teachers or students, use virtual reality for learning purposes, use their viewers less frequently, have tried less personal computer viewers and their viewers are not video games consoles (they usually prefer to approach virtual reality by means of mobile phones). These results can be explained considering that women usually approach virtual reality for working or learning purposes rather than a leisure one, which would justify the whole profile before described. These gender differences confirm the results obtained in recent studies, which still regard a greater consumption of digital leisure among men (Dindar, 2018; Rutherford, 2018).
• Concerning the educational level, apart from the differences between genders being significant, in cases with a higher educational level, there is higher relationship with formal education and the viewer is used less frequently. Taking into account that the main current use of virtual reality viewers is related to leisure (Dombrowski and Dombrowski, 2017; Sánchez-Cabrero et al., 2018a,b), this result is justified considering the previous conclusion, because an approach to this technology in a professional way implies a specific and unusual use of it.
• Regarding teachers and students (who are usually women with a high-level education), it can be asserted that they occasionally try advanced viewers, have mostly mobile viewers and use their viewers less often. Within these groups, as it is shown in this study, portability and easy access to this technology are more important than an advanced exploitation of its possibilities, which can be done through personal computer viewers. Moro's et al. (2017) study supports this assertion, giving a positive value to the educational efficiency of mobile viewers and its cost-performance ratio. Nevertheless, this study advised against the use beyond an occasional and unusual manner, as they found that mobile viewers caused dizziness and nauseas to a 40% of the studied sample (Moro et al., 2017).
• This study clearly confirms that those who try advanced virtual reality viewers on a computer finally acquire them and use them more frequently. Moreover, those individuals are not usually women or teachers. This result shows how important is to try the product for this new technology, because thanks to it, the potential buyer is encouraged to acquire the product. This conclusion is related to the study of Buń et al. (2017), who considered that one of the features hindering the expansion of virtual reality in the current market is the difficulty selling a product which can only be known through itself, which explains why the Internet, radio or TV commercials do not have the expected impact (Buń et al., 2017).
• If we analyse the results obtained under the variable ‘Number of years using virtual reality’, it can be observed that recent viewer's acquirers have not used them as a learning tool. Probably this could be mainly explained because they are still in a discovering stage of the technology (Kim et al., 2017) so they are initially interested in exploring its possibilities and living original experiences. Once this initial stage gets past, they will start to open their interest to those features that virtual reality can offer and value how virtual reality can provide them with what they really want.
• Finally, regarding the frequency of use of virtual reality, as it has already been presented under the previous variables, those who make a greater use are men, with lower educational level, no related to formal education and who have both tried and own advanced viewers. The results obtained by Dindar (2018) support the relationship shown in this study among gender, frequency of use and formative level.

In response to the second objective of this research, related to the assessment of the interest that early adopters of virtual reality may have in its use as a learning tool, in order to make an evaluation of its possible future inclusion within the formal educational environment, the following is concluded:

• First of all, the participants of the study directly related with formal education (students and teachers) are especially important in order to respond to this objective. They represent only the 23.1% of the sample and they prefer the portability that mobile virtual reality offers rather than the deeper experience that the others viewers can present, besides the fact that they only occasionally use this technology. These results partly explain why virtual reality implantation is being so slow in this field (Kavanagh et al., 2017). The possible future reduction in costs for the user of advanced virtual reality equipment may bring virtual reality closer to the classroom, as it can be stated from this study, because the use of an advanced VR viewer is directly associated with greater frequency of use. Currently, prices of viewers are excessively high in order to use them in the classroom in all their potential, so their use is very reduced and limited.
• Concerning the current use of virtual reality as a learning tool, it is still a minority who uses it for this purpose (13.7% of the sample). However, this fact is not yet alarming, because we are facing a technology that takes its first steps, with numerous lacks, especially regarding everything that goes beyond the leisure field (Domingo and Gates Bradley, 2017).
• Those who use virtual reality as a learning tool (13.7%) are usually women, show more interest in learning with virtual reality and would like to learn in the future in formal education using this technology. This particular low percentage is also explained because there are currently very few experiences available in this field and they have a very low budget, which makes that experiences related to other fields, such as videogames, video, tourism, etc. result much more attractive and better developed for the end user (Yildirim; 2017; Yildirim et al., 2018). Probably, the more the offer increases, the greater the percentage of users of virtual reality with an educational purpose will also be, as it is shown under higher percentages of other variables presented in this study such as ‘Interest in the use of virtual reality as a learning tool’ (28.2%), ‘Interest in the use of virtual reality in formal education in the future’ (51.3%) and ‘Optimism regarding the future pedagogical possibilities of virtual reality’ (47%).
• Regarding those who have interest in using virtual reality as a learning tool, in a first approach it could be asserted that the percentage is not so high. Nevertheless, when comparing them with those who currently use virtual reality viewers with pedagogical purposes, it clearly results that they are more than double (28.2% vs. 13.7%). This supports the idea that virtual reality is not often used as a learning tool due to the lack of experiences and applications, and not because users have no interest in it. On the other hand, there is a significant direct correlation between every variable related to the valuation of the virtual reality used as a learning tool, which explains the coherence regarding that interest in the future, when suitable conditions are in place.
• Finally, both variables ‘Interest in the use of virtual reality in formal education in the future’ (51.3%) and ‘Optimism regarding the future pedagogical possibilities of virtual reality’ (47%) show that a great percentage of users are interested in the virtual reality-education binomial. Therefore, we can be optimistic regarding its future evolution, as one of the more significant indicators associated to the consolidation of a product is the presence of an important number of individuals showing interest in it.

## 5 Conclusions

To sum up, according to the analysed results, it can be asserted that the relationship between the educational field and the virtual reality technology is currently at a crucial moment. This technology is now taking its first commercial steps and, therefore, its efforts are focused on putting itself in the map and promote its sales, mainly as a leisure tool, as the majority of applications designed for the different platforms and the greatest interest of their users are directed towards leisure (Newzoo, 2008; Statista, 2018). In this study, moreover, it has been observed that this marked interest in leisure is much greater regarding virtual reality viewers for video consoles (PSVR), which are especially designated for that use, and among male users who also use their computers more frequently. Its educational use is not a priority right now and those interested in it still have few options, as shown in the reduced supply of educational applications in the Oculus Store, for instance (Unimersiv, 2018). However, its current use is far from being a mere anecdote, as a 13.7% of use means a visible range of users of the whole.

Interest in the use of virtual reality as a learning tool is much higher than its current use, as concluded in this study, and the optimism concerning better pedagogical possibilities of virtual reality reaches almost half of the users. This rate of optimism, joined to the fact that this technology is taking its first steps and the initial conditions are not yet the best, shows indications rather encouraging. This conclusion is similar to other researches such as Yildirim's (2017) or Fernández-Robles (2016), who also observed an interest in the use of virtual reality as an educational tool among a sample of students.

Nevertheless, the progress of this relationship between education and virtual reality in the future depends on the development of applications and experiences within this concrete field. The results show that the lack of applications is hindering the interest of the users, so they are fundamental and necessary. Without them, these first green shoots could wither on short notice and this relationship could get cold and be wasted in the future.

Another problem to be faced in the future for the use of virtual reality as a learning tool is accessibility to groups of students. Currently, teachers participating in this study prefer cheap equipment and sporadic use. If prices are reduced, it is likely that teachers will end up using better equipment and increasing the time of use, considering greater possibilities of virtual reality as a tool for learning.

Discussions about the curricular quality and adequacy of schools to the 21st century reality are necessary as an urgent challenge with significant repercussion on the international political-pedagogical debate. Numerous organizations are urging upon the need of reformulating the pedagogical culture in order to achieve educational institutions where the ICT become actual pedagogical tools and, generally, the students could take advantage of their complete development, instead of been a mere mechanical response to problems disconnected from reality (Vivanco and Gorostiaga, 2017). At this point, virtual reality will play an important role, since it is the technology destined to bring the educational sphere closer to reality. It will be able to consider experiences so near to reality that they could generate emotions and sensations very similar to those generated by reality itself, bringing the distance between educational simulation and reality so close that they almost can touch each other.

The correct use of the virtual reality technology applied to the educational world is now part of these challenges. Software and all kind of tools and applications that make students travel to the inside of contents are spreading, allowing the reconstruction and direct experimentation of any imaginable situation inside the classroom (Costa-Roman, 2016). Virtual reality is the technology that will best achieve the objective of bringing the student closer to learning situations as close to reality, without the need to assume the risks involved (as, for example, travelling through the Milky Way, getting involved in a historical war, adopting the size of a molecule to know the microscopic world, etc). The possibilities of offering a greater quality education are multiplied exponentially, as well as the motivation of students towards learning (Fernández-Robles, 2016). So that, from all the new technologies applicable to education, there are good reasons to consider the development of virtual reality applications for education as a priority.

Nevertheless, due to their emerging pedagogical use, it is not currently possible to do a solid prospective reflection about every educational possibility that their correct use in teaching procedures would have. It will be necessary, at least, to wait until this technology settles down to evaluate the limits of its potential reach.

This study has described the social and demographic profile of the early adopters of virtual reality in Spain and has assessed their interest in this technology. Therefore, together with other studies, a series of rational evidences and considerations are emerging step by step that allows us to have a clearer and wider idea about the real possibilities of virtual reality that is arriving to the classrooms. To explore contexts, to experience sensations, to travel through time and to live experiences at the classroom, unthinkable a few years ago, it means a new world of possibilities that is modifying classrooms, educational institutions, pedagogical stereotype and, ultimately, the educational world as we know it until now.

## Declarations

### Author contribution statement

Roberto Sánchez-Cabrero: Conceived and designed the experiments; Performed the experiments; Analyzed and interpreted the data; Contributed reagents, materials, analysis tools or data; Wrote the paper.

Oscar Costa-Román, Francisco Javier Pericacho-Gómez, Miguel Ángel Novillo-López: Performed the experiments; Wrote the paper.

Amaya Arigita-García, Amelia Barrientos-Fernández: Analyzed and interpreted the data; Contributed reagents, materials, analysis tools or data.

### Funding statement

This research did not receive any specific grant from funding agencies in the public, commercial, or not-for-profit sectors.

### Competing interest statement

The authors declare no conflict of interest.

### Additional information

No additional information is available for this paper.

## References

[bib1] Artaud A. (1958). The Theatre and its Double. 1938.

[bib2] Bacos C., Carroll M. (2018). Kinematics for E-learning: examining movement and social interactions in virtual reality. Proceedings of E-Learn: World Conference on E-Learning in Corporate, Government, Healthcare, and Higher Education.

[bib3] Brooks F.P. (1999). What's real about virtual reality?. IEEE Comp. Graph. Appl..

[bib4] Buń P.K., Wichniarek R., Górski F., Grajewski D., Zawadzki P. (2017). Possibilities and determinants of using low-cost devices in virtual education applications. Eurasia J. Math. Sci. Technol. Educ..

[bib5] Cohen-Hatton S.R., Honey R.C. (2015). Goal-oriented training affects decision-making processes in virtual and simulated fire and rescue environments. J. Exp. Psychol. Appl..

[bib6] Costa-Román O. (2016). http://www.euskadi.eus/contenidos/informacion/dia6_biblioteca/es_bibliote/adjuntos/aldizkarien_berri_ekaina_2017/comunicacion_y_pedagogia_295_296.pdf.

[bib7] Dindar M. (2018). An empirical study on gender, video game play, academic success and complex problem solving skills. Comput. Educ..

[bib8] Disztinger P., Schlögl S., Groth A. (2017). Technology acceptance of virtual reality for travel planning. Information and Communication Technologies in Tourism 2017.

[bib9] Dockx K., Alcock L., Bekkers E., Ginis P., Reelick M., Pelosin E., Lagravinese G., Hausdorff J.,M., Mirelman A., Rochester L., Nieuwboer A. (2017). Fall-prone older people's attitudes towards the use of virtual reality technology for fall prevention. Gerontology.

[bib10] Dombrowski M., y Dombrowski J. (2017). Virtual reality games, therapeutic play and digital healing. International Conference on Distributed, Ambient, and Pervasive Interactions.

[bib11] Domingo J.R., y Gates Bradley E. (2017). Education student perceptions of virtual reality as a learning tool. J. Educ. Technol. Syst..

[bib12] Elotrolado.Net (2018). Foro de Sistemas VR in Multiplataforma. https://www.elotrolado.net/foro_multiplataforma-sistemas-vr_224.

[bib13] Enterteinment Software Association (2016). Essential Facts about the Computer and Video Game Industry. 2015: Sales, Demographic and Usage Data. http://www.theesa.com/wp-content/uploads/2015/04/ESA-Essential-Facts-2015.pdf.

[bib14] Fernández-Robles B. (2016). Factores que influyen en el uso y aceptación de objetos de aprendizaje de realidad aumentada en estudios universitarios de Educación Primaria. EDMETIC.

[bib15] Fowler C. (2015). Virtual reality and learning: where is the pedagogy?. Br. J. Educ. Technol..

[bib16] Gadelha R. (2018). Revolutionizing education: the promise of virtual reality. Child. Educ..

[bib17] Greenwald S., Kulik A., Kunert A., Beck S., Frohlich B., Cobb S., Parsons S., Newbutt N., Gouveia C., Cook C., Snyder A., Payne S., Holland J., Buessing S., Fields G., Corning W., Lee V., Xia L., Maes P. (2017). Technology and Applications for Collaborative Learning in Virtual Reality. http://eprints.uwe.ac.uk/32215/1/115.pdf.

[bib18] Heilig, M. L. (1962). U.S. Patent No. 3,050,870. Washington, DC: U.S. Patent and Trademark Office.

[bib19] Hockley L. (1997). Claudia Springer, Electronic Eros: Bodies and Desire in the Post-industrial Age.

[bib20] Instituto Nacional de Estadística (2017). Encuesta sobre Equipamiento y Uso de Información y Comunicación en los hogares 2017. http://www.ine.es/jaxi/Datos.htm?path=/t25/p450/base_2011/a2017/l0/&file=04001.px.

[bib21] Kavanagh S., Luxton-Reilly A., Wuensche B., Plimmer B. (2017). A systematic review of virtual reality in education. Themes Sci. Technol. Educ..

[bib22] Kim H., Fengfeng K., y Paek I. (2017). Game-based learning in an OpenSim-supported virtual environment on perceived motivational quality of learning. Technol. Pedagog. Educ..

[bib23] Leder J., Horlitz T., Puschmann P., Wittstock V., Schütz A. (2019). Comparing immersive virtual reality and powerpoint as methods for delivering safety training: impacts on risk perception, learning, and decision making. Saf. Sci..

[bib24] Lin M.T., Wang J., Kuo H., Luo Y. (2017). A study on the effect of virtual reality 3D exploratory education on students' creativity and leadership. Eurasia J. Math. Sci. Technol. Educ..

[bib25] Lowood H.E. (2015). Virtual Reality (VR). Encyclopaedia Britannica Online. https://www.britannica.com/technology/virtual-reality#%20ref884304.

[bib26] Luckey, P., Trexler, B. I., England, G., & McCauley, J. (2014). Virtual reality headset. U.S. Patent Application No 29/456,868.

[bib27] Menzies R.J., Rogers S.J., Phillips A.M., Chiarovano E., de Waele C., Verstraten F.A., MacDougall H. (2016). An objective measure for the visual fidelity of virtual reality and the risks of falls in a virtual environment. Virtual Real..

[bib28] Moro C., Stromberga Z., Stirling A. (2017). Virtualization devices for student learning: Comparison between desktop-based (Oculus Rift) and mobile-based (Gear VR) virtual reality in medical and health science education. Australas. J. Educ. Technol..

[bib29] Mütterlein J., Hess T. (2017). Immersion, presence, interactivity: towards a joint understanding of factors influencing virtual reality acceptance and use. https://aisel.aisnet.org/amcis2017/AdoptionIT/Presentations/17/.

[bib30] NEWZOO (2018). Newzoo's 2017 Report: Insights into the $108.9 Billion Global Games Market. https://newzoo.com/insights/articles/newzoo-2017-report-insights-into-the-108-9-billion-global-games-market/.

[bib31] Nissim Y., y Weissblueth E. (2017). Virtual reality (VR) as a source for self-efficacy in teacher training. Int. Educ. Stud..

[bib32] Oculus V.R. (2012). Oculus Rift: Step into the Game. Kickstarter. Sept, 1. https://www.kickstarter.com/projects/1523379957/oculus-rift-step-into-the-game?lang=es.

[bib33] Pan X., Hamilton A.F.D.C. (2018). Why and how to use virtual reality to study human social interaction: the challenges of exploring a new research landscape. Br. J. Psychol..

[bib34] Parong J., Mayer R.E. (2018). Learning science in immersive virtual reality. J. Educ. Psychol..

[bib35] Rizzo A., y Koenig S.T. (2017). Is clinical virtual reality ready for primetime?. Neuropsychology.

[bib36] Rutherford M. (2018). Video Gaming, Social Relationships, and Gender. https://digitalcommons.wou.edu/aes_event/2018/all/212.

[bib37] Sánchez-Cabrero R., Barrientos-Fernández A., Arigita-García A., Mañoso-Pacheco L., Costa-Román O. (2018). Demographic data, habits of use and personal impression of the first generation of users of virtual reality viewers in Spain. Data Brief.

[bib38] Sánchez-Cabrero R., Barrientos-Fernández A., y Maganto-Mateo C., Pérez-Fuentes M.C., Gázquez J.J., Molero M.M., Barragán A.B., Martos A., Simón M.M., Sisto (Comps.) M. (2018). La realidad virtual como recurso educativo para jóvenes visto a través de sus primeros usuarios. https://www.formacionasunivep.com/Vcice/files/libro%20avances%20de%20investigacion.pdf.

[bib39] SIMILARWEB (2018). Traffic Overview of Elotrolado.Net. https://www.similarweb.com/website/elotrolado.net.

[bib40] Statista (2018). Unit shipments of virtual reality (VR) devices worldwide from 2017 to 2018 (in millions), by vendor. Technology & Telecommunications›Consumer Electronics›Global Virtual Reality Device Shipments by Vendor 2017–2018.

[bib41] Stavova V., Dedkova L., Ukrop M., Matyas V. (2018). A large-scale comparative study of beta testers and regular users. Commun. ACM.

[bib42] Steuer J. (1992). Defining virtual reality: dimensions determining telepresence. J. Commun..

[bib43] Tussyadiah I.P., Wang D., Jung T.H., tom-Dieck M.C. (2018). Virtual reality, presence, and attitude change: empirical evidence from tourism. Tourism Manag..

[bib44] Unimersiv (2018). Educational Experiences/Apps for the Oculus Rift. https://unimersiv.com/reviews/oculus-rift/.

[bib45] Vivanco G., Gorostiaga J. (2017). Digital culture and diversity: perspectives of discourses on ICT-Education Policies. Cad. Pesqui..

[bib46] Wu W.N., Kuo F.Y.B. (2017). Play with google cardboard in a multiplayer environment: how do digital natives and digital immigrants differ?. https://aisel.aisnet.org/icis2017/HumanBehavior/Presentations/12/.

[bib47] Wu F., Liu Z., Wang J., Zhao Y. (2015). Establishment virtual maintenance environment based on VIRTOOLS to effectively enhance the sense of immersion of teaching equipment. Proceedings of the 2015 International Conference on Education Technology, Management and Humanities Science (ETMHS 2015).

[bib48] Yildirim G. (2017). The users' views on different types of instructional materials provided in virtual reality technologies. Eur. J. Educ. Studies..

[bib49] Yildirim G., Elban M., Yildirim S. (2018). Analysis of use of virtual reality technologies in history education: a case study. Asian J. Educ. Train..

[bib50] Zyda M. (2005). From visual simulation to virtual reality to games. Computer.

